# Proposition to Change the Organization of the Medical Association of Georgia

**Published:** 1886-10-20

**Authors:** 


### PROPOSITION TO CHANGE THE ORGANIZATION OF THE MEDICAL ASSOCIATION OF GEORGIA.

It will be remembered that at the last meeting of the Medical Association of Georgia, Dr. Wm. Abram Love, having at a previous meeting been appointed a fraternal representative to the Alabama Medical Association. made a report explanatory of, and favorable to, the adoption of the Alabama plan of organization. The Association of that State is conducted upon a new plan, and is thoroughly organized. Dr. Love speaks of the Association as unique and peculiar. The following extract from his report will serve to show the features which it is well the members of the profession should study, as it will be acted upon at the Georgia Medical Association to assemble in Atlanta, in April of next year. Of the Alabama Association he remarks:

"The membership consists in a College of Counselors and a body of Delegates. The College of Counselors consists of three classes or grades, and aggregates one hundred, nine of whom are elected annually. They rank as Junior Counselors, Senior Counselors, and Grand Senior Counselors. A proposition is now pending to establish a rank of Emeritus Grand Senior Counselors, into which to retire those who have faithfully served the Association through the subordinate positions to the expiration of their full terms of service.

"The body of Delegates consists of the regularly elected Delegates from the County Medical Societies—societies chartered by the Association under the State laws, giving authority for the same. Each County Society is entitled to two delegates exclusive of counselors. These represent the organized Profession of the several counties of the State.

"The officers of the Association consist of President, elected annually; two Vice-Presidents, elected for two years—one elected each year to fill the vacancy following a two-year's service of the senior; a Secretary, and a Treasurer, each elected for a term of five years; a Board of Censors and Committee of Public Health. This consists of ten members, elected from the College of Grand Senior Counselors, for a term of ten years—two each year, thus giving a large majority of experienced members on this important board, aside from being selected from among the Grand Senior Counselors—men who have reached that rank by virtue of having served satisfactorily five years as Junior Counselors, then promoted to the rank of Senior Counselors, served ten years as Seniors, when they are further promoted to the rank of Grand Senior Counselors, from which the Board of Censors is elected, as stated. This is an important board. It is composed of men true and tried—men of experience.

"While the Association constitutes the Board of Health of the State, under the enactments of its General Assembly, this Board of Censors constitutes the Executive Committee, or Acting Board *ad interim* for the Association in recess, the Senior Censor (chairman or president) being the Health Officer of the State.

"This body, as Censors, has the supervision of the County Medical Societies, and the revision of their rolls; the supervision of the County Examining Boards, and their examinations of applicants for license to practice medicine in the county or State. The examinations are conducted in writing, and the several applicants from irregular schools—as Eclectics, Homœpaths, etc.—are examined in Anatomy, Physiology, Chemistry, the Mechanism of Labor, Hygiene, and Medical Jurisprudence.

"Students desiring to enter upon the study of Medicine are examined as to their preliminary education, and, if not up to the standard, are not recommended. This, however, is only commendatory. Practitioners are examined on the several branches included in the curriculum of a college course, on Hygiene and Medical Jurisprudence, to which every graduate and undergraduate is subjected before he can legally practice medicine in the State. To guard against the influence of bias or prejudice in these examinations, and as a protection to both the County Examining Board and the applicant, these examinations are conducted in writing and sent up for the inspection of the Board of Censors."

